# Cerebral Microbleeds - To Treat or Not to Treat, That Is the Question: A Case Report With a Note on Its Radiologic Deconstruction and Therapeutic Nuances

**DOI:** 10.7759/cureus.10548

**Published:** 2020-09-20

**Authors:** Hassan Kesserwani

**Affiliations:** 1 Neurology, Flowers Medical Group, Dothan, USA

**Keywords:** mri images, cerebral vasculopathy, cerebrovascular diseases

## Abstract

With the ubiquity of susceptibility weighted imaging (SWI), cerebral microbleeds (CMBs) are fast becoming a prevalent phenomenon. They are tightly associated with age, neurodegeneration and diverse vascular etiologies. CMBs have a unique radiological signature. Their morphology, number and topology are quite informative. They also pose a therapeutic conundrum, as they are associated with the risk of cerebral hemorrhage. We present the case of an 86-year-old woman who has a vascular dementia, Binswanger's syndrome, and coronary artery disease, who presented with more than five CMBs. We present this case in order to highlight the dilemma of anti-platelet therapy in this group of patients and we demonstrate the cardinal radiologic features of CMBs. We then segue into the pathologic correlates of CMBs and associated risk factors. We finally analyze the risk of anti-platelet therapy in the presence of CMBs, and we unfold the latest data on CMB number and anti-platelet therapy.

## Introduction

Cerebral microbleeds (CMBs) are an increasingly recognized diagnostic entity. They represent microhemorrhages in brain parenchyma. Pathologically, these microbleeds are hemosiderin-laden macrophages. Deep seated microbleeds, in the corona radiata and basal ganglia, are more commonly seen in hypertension. Cortical lesions are commonly seen with amyloid angiography. Parasagittal linear streaks are more typical of brain trauma and diffuse axonal injury [1].

In the Rotterdam study of close to 4000 patients, CMB prevalence increases with age, from 6.5% in people aged 45 to 50 years to 35.7% in people older than 80 years. 15.3% of all patients had at least one CMB [2]. Systolic blood pressure, hypertension, smoking, lacunar infarcts and white matter lesions were associated with CMBs in a deep or infra-tentorial region, whereas apo-lipoprotein E4 (APO E4) and diastolic blood pressure were related to CMBs in a lobar location [2]. There is an elevated risk of both hemorrhagic, odds ratio 8.52 (4.23-17), and ischemic strokes, odds ratio of 1.55 (1.12-2.13), in patients with recent ischemic stroke or transient ischemic attacks (TIA) and CMBs [3].

Radiologically, CMBs appear magnified on susceptibility weighted images (SWI) due to their paramagnetic properties and this is known as a blooming artifact. CMBs appear dark, hypo-intense, on all SWI images. However, this finding can be due to either iron or calcium deposits. In order to differentiate, phase mapping is obtained [4]. These are rapid acquisition images at no extra cost. If the magnetic resonance imaging (MRI) scanner software is endowed with a left-handed reference frame such as Siemens, the CMBs appear hyper-intense on the phase map. If the MRI scanner software is endowed with a right-handed reference frame, the CMBs will appear hypo-intense on phase maps. To orient oneself, we can look at the superior sagittal sinus or a venous tributary, and establish the “color” of the MRI acquisition sequence on the phase map. In the left-handed reference frame, the sinus will appear bright. In the right-handed system such as a General Electric scanner, the CMBs appear dark by noting the dark sagittal sinus or a venous tributary. The reverse situation is seen in the left-handed Siemens scanner; here the CMBs and sagittal sinus appear bright.

SWI images and phase mapping are advanced imaging techniques that are sensitive to cerebral microbleeds. They utilize long echo sequences and gradient echo sequences (GRE). These image acquisitions rely on the magnetic susceptibility of tissues, whether paramagnetic or diamagnetic. Paramagnetism occurs when atoms have an odd number of electrons. Hence, they have a net magnetic dipole moment, which aligns and reinforces an applied magnetic field. If the number of electrons is even, that is paired, then there are no dipoles to align. However, the applied magnetic field distorts the motion of electrons via the Lorentz force. The induced magnetic dipoles anti-align with the applied magnetic field, and reduce the applied magnetic field. Such a material is diamagnetic. The filtered phase images or phase map exploit the magnetic properties of tissues; paramagnetic (such as iron) and diamagnetic (such as calcium), which have opposite signal intensities in phase mapping [5].

We present the case of an 86-year-old woman with Binswanger's disease characterized by a vascular dementia, lower half Parkinsonism and more than five CMBs. Binswanger's disease is a subcortical vascular dementia characterized by loss of executive function: planning, insight and foresight, with relative preservation of memory. It is frequently accompanied by a gait disorder. She has the typical risk factors of Binswanger's disease including chronic hypertension, coronary artery disease, hyperlipidemia and is a lifelong smoker. The question at hand states: is anti-platelet therapy safe in this patient with more than five micro-hemorrhages (CMBs) in the brain? The data to answer this question is evolving [6]. In this article, we lay out the pathologic correlates of CMBs, gently outline the radiological nuances involved in analyzing CMBs and finally we display the latest data addressing the role of anti-platelet therapy. As CMBs are incidentally discovered on routine MRI studies, whether to continue or discontinue anti-platelet or anti-coagulant therapy, is an open question. The clinician has to individualize therapy, weigh the benefits of therapy and consider the pros and cons, until more definitive data becomes available.

## Case presentation

We report the case of a previously active and independent 86-year-old divorced female patient who over the course of two years, especially last year, has become increasingly forgetful and confused. She left the stove on at least twice and once boiled eggs without water, forgets relevant family conversations and repeats questions frequently. She was no longer able to balance her checkbook and has become increasingly distracted, “spacey” and “not together”, as per her daughter's testimony. Her daughter also noted that she cannot keep up with conversations when there are more than two people conversing. Sometimes she cannot finish a sentence, has become increasingly argumentative and quit driving five years ago due to anxiety from a car accident. Her hygiene has declined, sometimes forgets to bathe or fix her hair, forgets to do laundry, but when remembers may wash clean clothes. In the past, she worked at convenience stores and focused on inventory. The patient now presents emotional lability with excessive crying, with abandonment of social activities, such as reading the newspaper and watching sitcoms.

Her medications include atorvastatin 20 mg, lisinopril 10 mg, escitalopram 10 mg, baby aspirin 81 mg and mirtazapine 30 mg daily. Her past medical history is significant for at least a 20-year-history of hypertension, coronary artery disease and hyperlipidemia. There is no family history of dementia. She has smoked cigarettes, at least one pack a day, for 50 years.

On examination, her blood pressure (BP) is 145/63 and a pulse of 67 beats per minute. Her height is five foot and three inches with a weight of 120 pounds and a body mass index (BMI) of 21.3. Precordial examination revealed no murmurs and carotid auscultation revealed no bruits. Her gait was stooped and shuffling, with adequate arm swing. No retropulsion was noted. At most, she had a mild symmetric bradykinesia but without a rest, postural or kinetic tremor. No sequence motion of the hands was noted with rapid finger apposition.

Cognitively she appeared distracted and a little paranoid despite a mini-mental state score (MMSE) of 27/30, which usually indicates a mild dementia when the score goes below 27. Visuo-motor skills were impaired with pantomime mimic. She has ideomotor apraxia with tool use, using the hand as the tool object. Deftness with a coin, limb-kinetic praxis, was preserved. Despite the presence of a grasp reflex and a snout reflex, a palmo-mental reflex was absent. But gegenhalten (paratonia) was noted in the arms, with compensatory increasing resistance with increasing velocity of flexion at the elbow.

Her cranial nerve examination was significant for a lively gag reflex. Power was preserved in the arms and legs, with symmetry. Her deep tendon reflexes were lively in the arms and legs, with a negative plantar extensor response bilaterally. Of note, stereognosis and graphesthesia were preserved in the hands.

**Figure 1:**
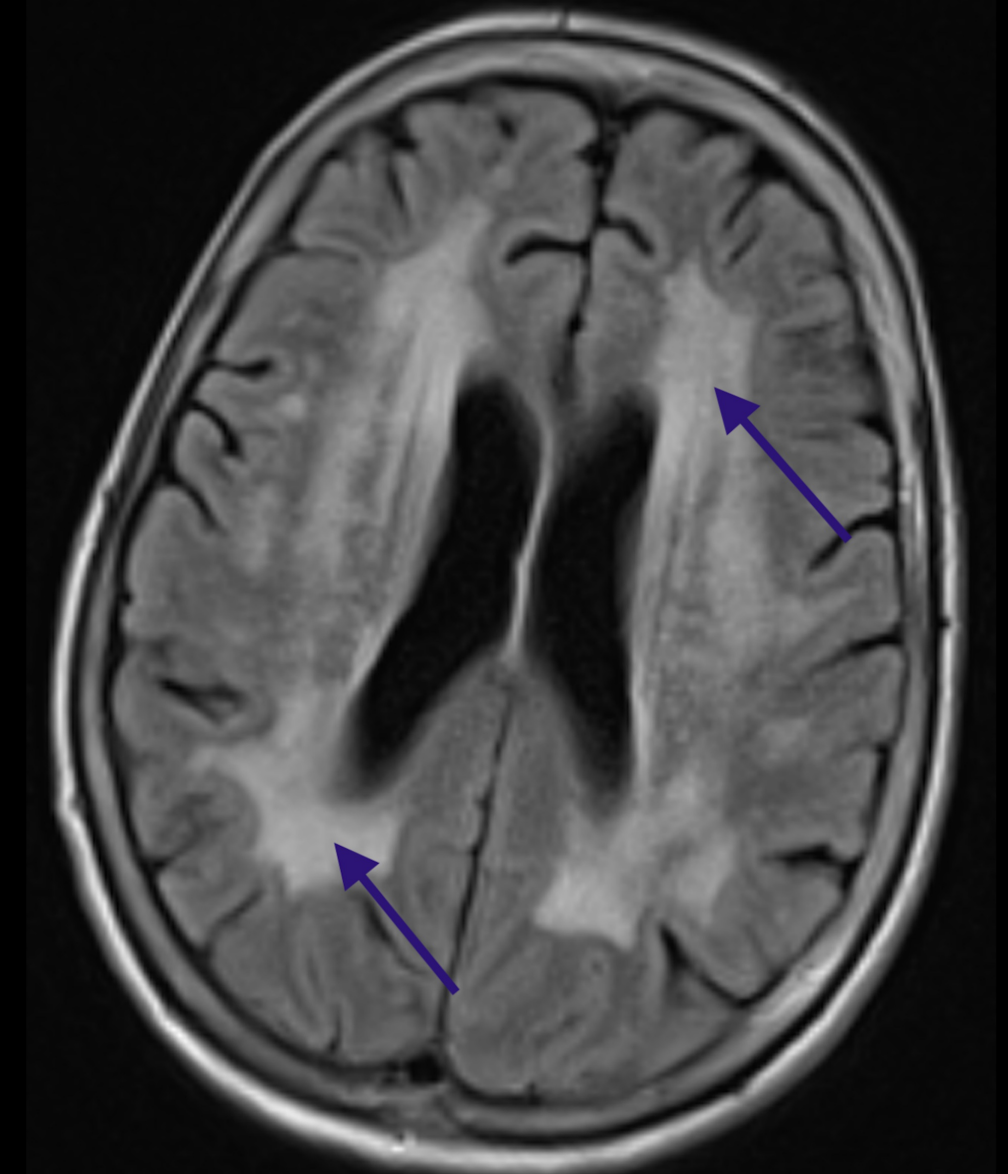
MRI of the brain: Demonstration of severe periventricular white matter disease, Binswanger's pattern (blue arrows) MRI: Magnetic Resonance Imaging

Due to her dementia presentation and clinical findings of lower half Parkinsonism, an MRI scan of the brain was obtained revealing severe subcortical white matter disease (Figure 1).

**Figure 2:**
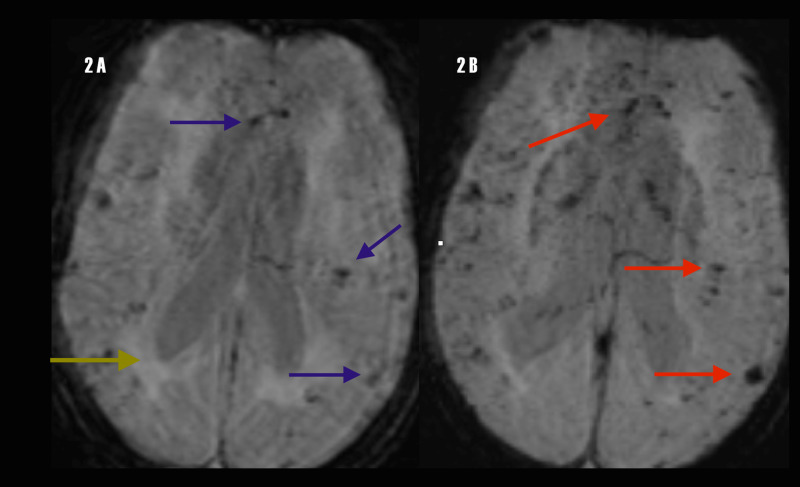
MRI: (A) Susceptibility weighted images (SWI) demonstrating hypointense CMBs (blue arrows). (B) Phase map revealing hypointense CMBs and betraying the right-handed reference frame of the MRI system (red arrows). These images look similar but are different acquisition sequences; note difference in peri-ventricular white matter intensity between both sequences (yellow arrow). MRI: Magnetic resonance imaging; CMB: Cerebral micro-bleeds; SWI: Susceptibility weighted images.

Susceptibility weighted images (SWI) and phase map images reveal cerebral micro-bleeds (MCBs), more than five lesions; note hypo-intensity of bleeds on both sequences implying a right-handed reference frame (Figure 2).

A carotid duplex scan and a transcranial Doppler ultrasound did not reveal any significant cervical carotid or intra-cranial artery stenosis respectively. She has stable coronary artery disease and more than five CMBs on susceptibility MRI of the brain. Hence one faces a therapeutic dilemma; with such extensive cerebral white matter disease and stable coronary artery disease, is anti-platelet therapy warranted in the presence of more than five CMBs? Our solution was to continue the statin, atorvastatin 20 mg and aspirin 81 mg daily for the following reasons. We considered substituting cilostazol, an anti-platelet phosphodiesterase inhibitor for aspirin, as we do know that when cilostazol is compared with aspirin, the risk of cerebral and gastrointestinal hemorrhage is lower, with a 25.7% relative risk reduction of ischemic strokes, but only in patients with prior ischemic strokes [7]. However, there is no data for the effectiveness of cilostazol in stable coronary artery disease. We decided to continue low dose aspirin therapy in order to protect her coronary arteries. We continued statin therapy, atorvastatin 20 mg daily, as this too has anti-thrombotic effects on both the coronary and cerebral circulation. She was encouraged to quit smoking. For her cognitive impairment, the patient was started on an acetylcholine-esterase inhibitor, donepezil 5 mg daily. The risk of an ischemic or hemorrhagic stroke in patients with less than five or more than five CMBs, treated with anti-platelets, will be outlined in the Discussion section.

## Discussion

As outlined earlier, CMBs are radiologically small round or ovoid regions of signal loss seen on paramagnetic MRI sequences. CMBs are due to hemosiderin-laden macrophages. CMBs are a direct result of extravasation of erythrocytes from diseased arterioles and capillaries damaged by hypertension, such as the small perforating arteries of the deep gray and white matter, in the basal ganglia and the lobar regions. In Caucasians with intracerebral hemorrhage, CMBs in a lobar distribution are associated with cerebral amyloid angiopathy (CAA). CAA leads to progressive deposition of β-amyloid in small cortical and leptomeningeal arterial walls, increasing their fragility [1,3].

Lipohyalinosis, also known as fibrinoid necrosis, occurs in small vessels in hypertensives, affecting deep perforating arteries, which branch off from large arteries in the basal ganglia, white matter, brain stem and cerebellum. The fibrinoid deposition in the tunica media of the blood vessel wall is due to blood-brain barrier disruption leading to destruction of smooth muscles and the extracellular matrix. This leads to the formation of micro-aneurysms and micro-hemorrhages. Hyaline arteriolosclerosis is characterized by thickening of the arteriolar wall by collagen deposits around the basement membrane, leading to fragility and CMBs [8,9]. The pathologic and radiologic findings of CMBs are summarized below (Table 1).

**Table 1:** Morphology, pathology and radiologic characteristics of CMBs CMB: Cerebral microbleed; GRE: Gradient echo imaging; SWI: Susceptibility weighted imaging.

MORPHOLOGY OF CMB	PATHOLOGY	RADIOLOGIC CHARACTERISTICS
Size of lesion < 10 millimeters; round or ovoid	Microbleed - extravasation of erythrocytes	Hyperintensity on GRE/SWI
Location depends on etiology	Hemosiderin-laden macrophages	Blooming artefact on SWI
	Vessel diameter < 200 micrometer	Hypointense or hyperintense on phase map
	Lipohyalinosis of blood vessel wall	
	Amyloid deposition on vessel wall	

In patients with cerebral amyloid angiopathy (CAA), the prevalence of CMBs is 100%. The distribution of CMBs in CAA is lobar, mirroring the histopathology of amyloid angiopathy in cortical vessels. This lobar pattern is also seen in Alzheimer's disease. Involvement of the leptomeningeal vessels explains the occurrence of superficial siderosis that is observed in both CAA and Alzheimer's disease [10]. CMBs also occur more frequently in patients with vascular dementia displaying a more central distribution pattern, and may involve the thalamus, brainstem and cerebellum [11]. In diffuse axonal injury, CMBs are typically located in the corpus callosum and at the gray-white matter junction, and tend to have a more radial configuration following the perivascular spaces compared with the more spherical CMBs occurring with neurodegeneration or hypertension [12].

Radiation vascular injury begins above a dose of 25 Grays (Gy). It can acutely involve the small vessels, through fibrinoid necrosis and telangiectasis with vessel permeability and vasogenic edema. Chronically, the larger vessels are affected with vessel wall thickening, thrombosis and fibrinoid necrosis. CMBs occur in at least 50% of patients who have undergone radiation treatment, in pediatric and adult patients. The rate of CMB formation increases significantly two years after radiation treatment and is associated with cognitive impairment [13-15].

Cavernous angiomas have a popcorn-like high signal intensity on T1-weighted images and a hypo-intense hemosiderin ring on T2-weighted images. Small type IV cavernous angiomas may be indistinguishable from CMBs. Cavernous angiomas can be classified into four types: type I - extra-lesional blood beyond cavernous angioma; type II - mixture of subacute and chronic blood; type III - area of hemosiderin with a small central core; and type IV - area of hemosiderin deposition without a central core. Because of a lack of a central core, type IV lesions are only visible on SWI images as hypo-intense spots, identical with CMBs [16], type IV lesions being the most similar to CMBs.

SWI hypo-intensity and T1 hyper-intensity have been deployed to detect micro-metastases. These signal characteristics are five times more common in melanoma metastases than in lung cancer metastases. Three quarters of melanoma metastases have one or the other signal characteristic and a quarter have both findings. This combination of findings was sixteen times more common with melanoma metastases than with lung cancer metastases. Melanin leads to T1 shortening and the propensity of melanomas bleeding, with methemoglobin accumulation, can lead to T1 shortening. The susceptibility effects on SWI are due to the presence of metal ions: iron, copper, manganese, and zinc. Of note, 7% of melanomas had no T1 shortening and were only detected with SWI [17].

There are strong associations of CMBs with age and hypertension, more likely deep in the brain. Carrying the apo-lipoprotein E4 (APO E4) gene increases the risk of lobar CMBs most frequently in the parietal lobes [2]. CMBs are strongly correlated with volume of white matter disease [18].

The presence and number of CMBs also correlates with the congestive heart failure/left ventricular systolic dysfunction, hypertension, age ≥75 years, diabetes mellitus, stroke or TIA or thromboembolism, vascular disease (CHADS2-VASC) scores, which is used to estimate ischemic stroke risk in patients with atrial fibrillation. In a study of 131 Japanese patients with atrial fibrillation and 112 controls without atrial fibrillation, patients with atrial fibrillation had a significantly higher prevalence of CMBs. There is also evidence that lobar CMBs may be more common than deep CMBs in patients with atrial fibrillation [19].

The various causes of CMBs with their topography and mechanisms are listed below (Table 2).

**Table 2:** Risk factors associated with CMBs CAA: Cerebral amyloid angiopathy; Covid-2019: Corona virus disease 2019; CMB: Cerebral microbleed.

STUDY	RISK FACTOR	LOCATION OF LESION	MECHANISM
Knudsen et al. [10]	CAA / Alzheimer's disease	Cortical - sub-cortical - lobar	Amyloid angiopathy / superficial siderosis
Vernooij et al. [11]	Hypertension / Vascular dementia	Thalamus - brainstem - cerebellum (deep and infra-tentorial)	Lipohyalinosis in deep perforating blood vessels
Imaizumi et al. [12]	Head trauma	Parasagittal, corpus callosum, white matter-gray matter junction	Diffuse axonal injury
Tanino et al. [14]	Radiation necrosis	Variable	Fibrinoid necrosis / thrombosis
Schmidt et al. [18]	White matter disease	As in hypertension above	As in hypertension above
Saito et al. [19]	Atrial fibrillation	Lobar	Micro-emboli
Fitsiori et al. [20]	Covid-19	Corpus callosum, internal capsule, middle cerebellar peduncle	Inflammatory - cytokine cascade / thrombosis

In the study by Lau et al., in 1811 patients who were prescribed anti-platelet therapy, the five-year risk of recurrent ischemic stroke and hemorrhagic stroke both increased with the number of CMBs [6]. High CMB burden, greater than or equal to five, was an independent risk factor for recurrent ischemic stroke, hemorrhage stroke, all cause mortality, and nonvascular death.

Patients with greater than or equal to five CMBs experienced a three-fold higher risk of recurrent ischemic stroke and a 13-fold increased risk of hemorrhagic stroke than those without CMBs. For the patients with less than five CMBs, the five-year absolute risk of ischemic stroke was much higher than the incidence of intracranial hemorrhage. For those with greater than or equal to five CMBs, the risk of fatal and disabling ischemic and hemorrhagic strokes was similar in the first year, but this calculus changed from the second to fifth year, where the risk of a hemorrhagic stroke was much higher, almost 13 times higher [6].

In summary, CMBs are associated with a higher risk of ischemic strokes than hemorrhagic strokes during the first year, regardless of the number of CMBs and treatment with anti-platelets is recommended, if indicated. For more than or equal to five CMBs, this issue is still contentious, as a head to head study is not available. However, withdrawal of anti-platelet therapy may be considered depending on the risk profile. These findings are summarized below (Table 3).

**Table 3:** Summary of study by Lau et al. Findings suggest that anti-platelet use is safe when CMB number is below five, but questionable when CMB number exceeds five from year two to year five [6]. CMBs: Cerebral microbleeds

	< 5 CMBs	> 5 CMBs
YEAR ONE	Ischemic stroke exceeds hemorrhagic stroke rate	Ischemic stroke risk exceeds hemorrhagic stroke risk
YEAR TWO TO FIVE	Ischemic stroke exceeds hemorrhagic stroke rate	Hemorrhagic stroke far exceeds ischemic stroke
USE OF ANTI-PLATELET THERAPY	Yes: year one to five	Yes: year one. Questionable: year two to five

## Conclusions

Our case highlights the dilemma the clinician faces in patients who carry more than five CMBs on an MRI of the brain and who have co-existent co-morbid diseases, such as coronary artery disease. As we highlight in the Discussion section, anti-platelet therapy in patients who harbor more than five CMBs can be associated with as high as a 13-fold increased risk of an intracranial bleed, especially in those patients who are receiving anti-platelet therapy for more than one year. We tackle this therapeutic conundrum by considering an alternative therapy with cilostazol, a phosphodiesterase inhibitor, which is less likely to lead to an intracranial bleed. However, this approach was deemed insoluble, as there is no evidence for the effectiveness of cilostazol for coronary artery disease. The literature of CMBs is extensive. In this article, we streamline a lot of the data and address the key points. In particular, we unravel the radiological signature of CMBs, its pathophysiologic correlates, outline the risk factors and common diseases associated with CMBs and at the end of the discussion we address the risk of ischemic and hemorrhagic strokes in patients with CMBs who are receiving anti-platelet therapy.
